# Book Review

**DOI:** 10.1120/jacmp.v7i4.2418

**Published:** 2006-11-28

**Authors:** Joel E. Gray

**Affiliations:** ^1^ Consultant & President DIQUAD, LLC

## Abstract

*Physics for Radiation Protection: A Handbook*, James E. Martin, PhD, CHP, Editor, John Wiley Publishers, ISBN 3527406115, $250 Hc

This is an extensive treatise on physics for radiation protection and a completely revised and updated second edition. The author refers to the book as “A highly practical resource for health physicists and other professionals in radiation protection” and, indeed, it is. At 822 pages (and in 10 point type) there is a lot of valuable information between the covers. This can serve as either a handbook or a textbook. The author indicates that a course in radiation physics would be based on material in 7 chapters “with selections from other chapters, all or in part,” addressing specialty areas of interest to the instructor and student.

The handbook includes 15 chapters and 6 appendices, including Atoms and Energy, Major Discoveries in Radiation Physics, Radioactive Transformation, Interactions, Nuclear Fission and Its Products, Naturally Occurring Radiation and Radioactivity, Interactions of Radiation and Matter, Radiation Shielding, Internal Radiation Dose, Nuclear Criticality, Radiation Detection and Measurement, Statistics in Radiation Physics, Neutrons, and X Rays. Each chapter is well written in an understandable style. This book includes 400 illustrations, many problem examples, and sample problems on which students can hone their skills.

Chapter 8 on Radiation Shielding covers shielding of alpha‐, and beta‐emitting sources, photon sources, the effect of the buildup on shield thickness; line, disc and planar, and area sources; shielding of protons and light ions, among other topics. However, one could not shield a diagnostic or therapeutic x‐ray room based on the information provided. The text (copyrighted in 2006) references NCRP 51, “Radiation Protection Design Guidelines for 0.1 – 100 MeV Particle Accelerator Facilities,” published in 1977, but does not mention the recently published NCRP Reports 147 and 151 on structural shielding design of diagnostic and therapeutic facilities. A section on practical shielding design in medical physics would be a useful addition for medical physicists.

Chapter 15 discusses x‐ray shielding and reproduces many of the curves from NCRP Report 49, published in 1988. As noted above, recently published NCRP Reports 147 and 151 are available and supersede NCRP 49.

Another oversight is the omission of optical stimulated luminescence (OSL) from the section on Radiation Detection and Measurement. Film badges, TLDs, and pocket dosimeters are discussed. However, OSL dosimetry is not even though about 75% of radiation workers in the U.S. are on OSL dosimetry and about 1.5 million OSL personnel dosimeters are produced monthly for use worldwide.

Despite these few weaknesses *Physics for Radiation Protection* is an excellent book. It contains a significant amount of valuable information and will serve as an excellent reference on the bookshelf of medical physicists. However, one should be forewarned that the price is $250!

